# Micropipette-Based Microfluidic Device for Monodisperse Microbubbles Generation

**DOI:** 10.3390/mi9080387

**Published:** 2018-08-04

**Authors:** Carlos Toshiyuki Matsumi, Wilson José da Silva, Fábio Kurt Schneider, Joaquim Miguel Maia, Rigoberto E. M. Morales, Walter Duarte Araújo Filho

**Affiliations:** 1 Department of Electronics, Federal Institute of Education, Science and Technology of Santa Catarina (IFSC), Joinville, SC 89220-618, Brazil; 2 Graduate Program in Electrical and Computer Engineering (CPGEI) and Electronics Engineering Department (DAELN), Federal University of Technology Paraná (UTFPR), Curitiba, PR 80230-901, Brazil; fabioks@utfpr.edu.br (F.K.S.); joaquim@utfpr.edu.br (J.M.M.); 3 Graduate Program in Mechanical and Material Engineering (PPGEM) and Department of Mechanics (DAMEC), Federal University of Technology Paraná (UTFPR), Curitiba, PR 80230-901, Brazil; rmorales@utfpr.edu.br; 4 Department of Exact and Earth Sciences (DCET), University of the State of Bahia (UNEB), Salvador, BA 41150-000, Brazil; wfilho@uneb.br

**Keywords:** 3D printing microfluidic devices, microbubbles, micropipettes, cross-flow

## Abstract

Microbubbles have various applications including their use as carrier agents for localized delivery of genes and drugs and in medical diagnostic imagery. Various techniques are used for the production of monodisperse microbubbles including the Gyratory, the coaxial electro-hydrodynamic atomization (CEHDA), the sonication methods, and the use of microfluidic devices. Some of these techniques require safety procedures during the application of intense electric fields (e.g., CEHDA) or soft lithography equipment for the production of microfluidic devices. This study presents a hybrid manufacturing process using micropipettes and 3D printing for the construction of a T-Junction microfluidic device resulting in simple and low cost generation of monodisperse microbubbles. In this work, microbubbles with an average size of 16.6 to 57.7 μm and a polydispersity index (PDI) between 0.47% and 1.06% were generated. When the device is used at higher bubble production rate, the average diameter was 42.8 μm with increased PDI of 3.13%. In addition, a second-order polynomial characteristic curve useful to estimate micropipette internal diameter necessary to generate a desired microbubble size is presented and a linear relationship between the ratio of gaseous and liquid phases flows and the ratio of microbubble and micropipette diameters (i.e., Q_g_/Q_l_ and *D*_b_/*D*_p_) was found.

## 1. Introduction

In recent years, there has been a growing interest in the application of microbubbles (i.e., as bubbles in the range of 1–1000 μm) in various fields of medicine, pharmacology, and chemistry, as well as in the food industry [1]. Sizes vary according application. In water treatment, microbubbles ranging from 10 to 100 μm are required [2]. In diagnostic medical imaging and for gene and drug delivery, the microbubbles required are usually in the range of 2–8 μm [3]. Microbubbles are composed of a gaseous core and an outer coating layer, which may consist of various materials, including human albumin, phospholipids, surfactants, and other compounds [4,5,6,7,8].

There are several techniques to generate microbubbles including the Gyratory method and coaxial electro-hydrodynamic atomization (CEHDA), as well as the use of sonication and microfluidic devices [1,9,10,11]. Sonication, is a commonly used method for preparing microbubbles through the dispersion of gas or liquid in a suspension of a suitable coating material using high intensity ultrasound [12]. The gas or liquid is emulsified to form a suspension of bubbles in high temperatures and pressures generated as a result of inertial cavitation in the suspension. The bubble size distribution depends on the frequency, power, and pulse of the ultrasound [5,12]. The production of microbubbles by coaxial electro-hydrodynamic atomization (CEHDA), evolved from conventional electrohydrodynamic atomization [13]. Its configuration is based on the existence of two concentrically aligned coaxial channels, the internal channel being driven by a gas and the external channel per liquid. An electric field is applied between the outer channel and a grounded collector base, with sufficient intensity to exceed the surface tension threshold of the liquid, forming a cone from the channel mouth from which a very fine jet emerges. This jet breaks down forming droplets associated with microbubbles. The continuous and uniform generation of microbubbles depends on the combination of gas flow parameters adjustment, liquid flow, applied electric field, viscosity, and surface tension of the liquid [10,13,14]. Farook et al., (2007), using the CEHDA technique, produced microbubbles with diameters of 2 to 8 μm and described that the size of microbubble distribution critically depends on the relationship between liquid/air flows and, in particular, the air flow rate [9]. In 2008, Stride and Edirisinghe carried out a work comparing microbubble preparation techniques. The techniques were sonication, coaxial electrohydrodynamic atomization, and microfluidic processing (T-junction). They analyzed the rate of bubble production, stability, mean diameter, and standard deviation. The T-junction technique produced microbubbles with a polydispersity index (PDI) of approximately 1%, for the CEHDA and sonication techniques PDI of 30% and 150% were found, respectively [10]. In 2014, Parhizkar, Stride, and Edirisinghe have shown a microfluidic setup combined with electrohydrodynamic processing of bubble formation at different applied voltages and with different liquid properties. In their experiments they observed that the diameter of the bubble decreased dramatically with the increase of the voltage between 0 and 9 kV, for solutions with viscosity between 1.3 mPa·s and 36 mPa·s. With the viscosity of 1.3 mPa·s, the bubble size without application of the electric field was 170 μm and 40 μm with the electric field at 9 kV, obtaining a low PDI of 1% [15]. The gyratory method is a technique that uses dynamic fluid flow and centrifugal force to jet out microbubbles consistently. Careful selection of operating conditions (i.e., speed of rotation and the working pressure of the solution) is vital to the success of the process due to the minimum pressure threshold and a minimum rotational velocity for bubble formation, below this threshold the bubbles are not formed due to insufficient fluid flow or low centrifugal velocity [11].

In microbubble generation by microfluidic devices, the most commonly used models are the coflow, the cross-flow or T-junction, and the flow-focusing [16,17,18,19]. For the coflow configuration, the gas is supplied in the same direction as the liquid. The flow of the liquid is external to the gas flow, squeezing the gas and generating the bubbles. In the coflow technique, the bubble size increases proportionally to the increase in viscosity of the continuous phase, but independent of surface tension and flow rates (Yasuno et al., 2004) [20]. In the flow-focusing technique, the liquid and gas flows are transported through different channels and forced to flow simultaneously through a narrow constriction channel, i.e., focusing channel, with a determined diameter located downstream of the gas injector. The constriction of the channel produces an axial pressure gradient, where the gas is squeezed by the liquid, creating bubbles after passage through the narrow channel. In 2001, Gañán-Calvo and Gordillo described the formation of monodisperse gas bubbles in capillaries, describing that bubble size formation mainly consists of the ratio of the gas flow to the liquid flow, and is independent of the viscosity, surface tension, and Reynolds number [19]. On the other hand, Garstecki et al., (2004), described a flow-focusing device embedded directly in a microfluidic chip with polydispersity index smaller than 2% reporting that viscous effects are important in the formation of bubbles [18]. Hettiarachchi et al. (2007), fabricated a similar device using soft lithography achieving high bubble stability and production rate in the order of 6 × 10^7^ bubbles per minute, with a PDI around 2% [21]. Peyman et al. (2012), introduce a new device where bubbles were formed outside and upstream of the nozzle improving the rate of microbubble production showing highly reproducibility capable of producing 10^10^ bubbles mL^−1^ in 10 min [22]. The cross-flow (i.e., T-Junction) device configuration, two channels are arranged perpendicularly and converge to a common point. One of the channels is fed by the liquid phase and the other by the gas phase. Due to the instability of the interface between gas and liquid, the gas is squeezed by the liquid at the point wherein the channels meet, leading to the generation of the microbubbles [23,24]. Depending on the speed of the compression process, microbubbles of different sizes can be produced. This technique to generate microbubbles directly depends on the composition of the liquid phase, the gas composition, the pressure, the viscosity of the liquid phase, and the width of the channel that feeds the gas phase [25,26,27]. Garstecki et al. (2006), show that the dominant contribution to the rupture dynamics arises from the pressure drop across the emerging bubble. The equation for the length of the immiscible slug L, is proportional to the flow rate ratio in T-junction: L/d = 1 + α(Q_g_/Q_l_) where d is the width of the channel, Q_l_ and Q_g_ are the liquid and gas flow rates, respectively, and α is a constant [17]. Pancholi et al. (2008), investigated the dynamics of bubble formation in highly viscous liquids in a T-junction device. They determined the influence of different parameters on bubble size processing for different liquid and gas flow rates (Q_l_/Q_g_) and in particular the viscosity of the liquid. In their theoretical and experimental results showed that the viscosity of the liquid and the flows of liquid and gas had a significant influence on the formation of bubbles and their size, with higher viscosities and higher flow rates producing smaller bubbles [24]. Jiang et al. (2016), describe a new device composed of two T-junctions arranged in series with coarse capillaries. The microbubbles produced through a double T-junction were smaller in size and more stable compared to a single T-junction based device for the same parameters of viscosity, liquid flow, and gas pressure. According to their predictive model, microbubbles with a diameter of 200 μm can be reduced, through the usage of several junctions, to diameters smaller than 10 μm [3]. According to Stride and Edirisinghe, the T-junction technique can generate microbubbles in a single step, with a PDI of 1% at lower operational costs [10,24].

In our previous work, we introduced a low cost hybrid device composed of micropipette inserted into a microchannel device created using 3D printing capable of producing biocompatible microbubbles with minimum size of 73.7 µm [25]. In this work, we present the production of smaller microbubbles and a study on microbubble production linearity in relation to gaseous and liquid phase flow as well as an experimental-based estimation curve correlating the micropipette internal diameter as a function of the microbubble diameter considering a given emulsion and various gaseous and liquid phase flow values.

## 2. Materials and Methods

**Figure 1:**
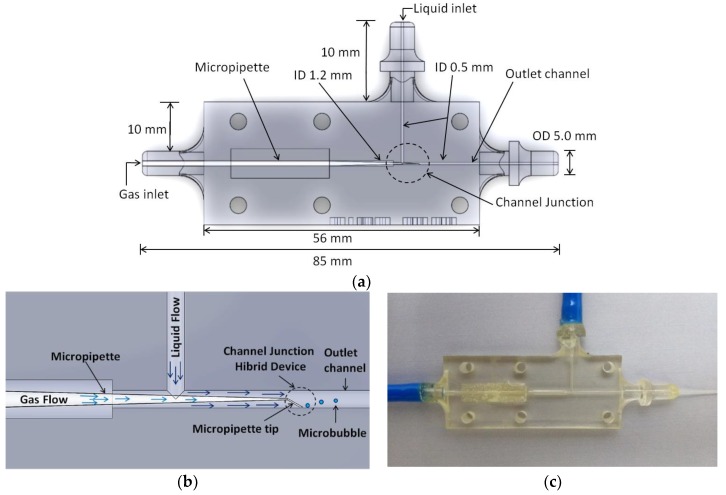
Hybrid microfluidic device developed in a 3D printer. In which: (**a**) represents the device developed with the technical dimensional specifications, (**b**) represents the trunk of the channel with the tip of the micropipette inserted into the device, and (**c**) the actual device with the incorporated micropipette.

The developed T-Junction devices were fabricated in the 3D Objet Eden 250 (Stratasys, Eden Prairie, MN, USA) rapid prototyping machine using a transparent resin (Fullcure 720, Stratasys, Eden Prairie, MN, USA) at the Prototyping and Tooling Center (NUFER, UTFPR laboratory, Curitiba, Brazil). This resin is used especially for the manufacture of a wide variety of rigid prototypes, wherein the visualization through the prototype wall is necessary. Figure 1 illustrates the hybrid microfluidic device with a micropipette inserted, where (a) represents the device developed with the technical dimensional specifications, (b) represents the trunk of the channel with the tip of the micropipette inserted into the device, and (c) the actual device with the incorporated micropipette.

**Figure 2:**
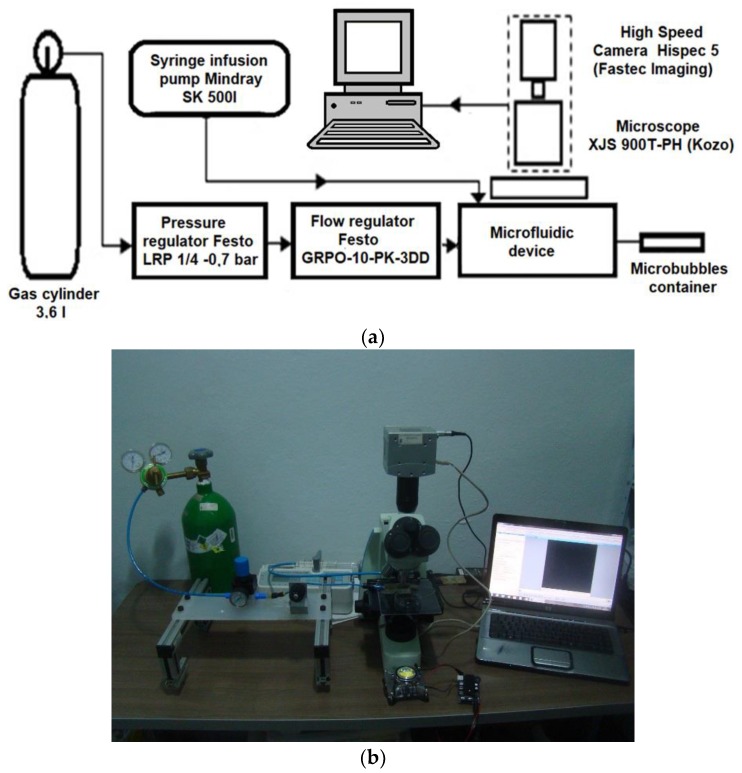
(**a**) Diagram of the experimental apparatus used in the generation of microbubbles. (**b**) A picture of the actual experimental setup.

The diagram of the experimental microbubble generation apparatus is shown in Figure 2a and a picture of the actual experimental setup is shown in Figure 2b. A working video is available in the ESI†: S1_Video Experimental apparatus.mpg. The gas phase was supplied by a 3.6 L gas cylinder, with a conventional manometer reader with maximum pressure reading of 15 bars coupled to its output. The cylinder was filled with compressed air with approximately 30% of oxygen and 70% of nitrogen. The gaseous phase control is done via the precision pressure regulator model LRP 1/4-0.7 (Festo, Esslingen, Germany), with a maximum inlet pressure of 12 bars and a maximum outlet pressure of 0.7 bar. This regulator was coupled to a conventional pressure gauge, enabling simplified readings during necessary adjustments of the gaseous flow correction. It was also coupled to a precision flow regulator valve GRPO-10-PK-3DD from Festo. The two regulators are manually adjustable by a scaled rotary handle. Lastly, these regulators are connected to the junction microfluidic device. The liquid phase flow control was performed through a syringe-based infusion pump SK-500I (Shenzhen Mindray Bio-Medical Electronics, Shenzen, China). The flow is digitally adjusted from 1.67 µL·min^−1^ to 8333.33 µL·min^−1^ using 10 mL, 20 mL, and 50 mL syringes.

The bubble coating was composed of Tween 80 surfactant associated with a lipid due to the biocompatibility characteristics and the ability to generate more echogenic microbubbles, compared to polymer coatings [26]. The lipid used was sunflower oil. The air was the gaseous component of the microbubble. The microbubble generation process was monitored using a XJS-900T-PH (Nanjing Kozo Optical and Eletronical Instruments, Nanjing, China) microscope with a 20× magnification objective lens, coupled to a Hispec 5 digital camera (Fastec Imaging, San Diego, CA, USA), allowing total resolution of 1696 × 1710 pixels and 523 frames per second (fps). The camera has enough storage for collecting a sequence of for later data transfer to a computer for processing. The microbubbles generated were collected and stored in a container with deionized water.

An algorithm was developed to evaluate the microbubble diameter from high frame rate videos by the Multiphase Flow Research Center (NUEM, UTFPR laboratory, Curitiba, Brazil) and implemented using MATLAB (MATHWORKS, Natick, MA, USA). To determine the diameter of the microbubbles, the images are segmented by subtractive techniques where a reference image without microbubble is subtracted from an image under analysis. The segmented image shows the microbubble region with white pixels on a dark background. The number of pixels is counted automatically for each closed region. In order to define the actual area, pixel-count is done using a micrometric scale. In addition to automatic size determination through that tool developed on MATLAB, the diameter of the generated microbubble was also evaluated using the digital camera coupled to a 4.3× magnification microscope and a 1 DIV/10 μm micrometer ruler scale.

The liquid phase used to feed the system consisted of an emulsion composed of 98 wt. % of deionized water, 1 wt. % of sunflower oil, and 1 wt. % of Tween 80 surfactant, viscosity (µ) of 1.17 mPa·s value obtained with a Brookfield DV-E rotary viscometer, surface tension of 0.063 N·m^−1^ at 21 °C and local atmospheric pressure of about 91 kPa at NUEM-UTFPR. The measured surface tension of the fluid, using Tate’s law, was 0.063 N·m^−1^. For the surface tension definition we used 10 experiments measuring 50 drops in each experiment.

In a microchannel device, the hydrodynamic property for the flow is characterized by the low values of Reynolds number, where viscous forces are dominant. When Re << 1, the flow is dominated by viscous stress and surface tension [17]. The Reynolds number was found using Re = Q_l_ρ_1_*D*_ch_/µ, where μ is the viscosity, Q_l_ is the liquid flow, ρ_1_ is the density of liquid, and *D*_ch_ is the hydraulic flow diameter of the circular pipe. The Reynolds number in our experiments was found to be in the range 0.047 < Re < 0.058. The inertia force can be neglected and the capillary number is the important dimensionless parameter for predicting the flow regime of bubbles’ generation. The capillary number for the liquid was found using Ca = µU_l_/σ, where σ is the surface tension and U_l_ is the liquid velocity. At very low Ca number, the film thickness of the gas bubble is negligible. The capillary number in our experiments was found to be in the range of 0.0017 < Ca < 0.0021, therefore, the breakup mechanism of microbubble formation is found to be in the squeezing regime [3]. The fluid velocity in our work was in the range 11,035 µm·s^−1^ < U_l_ < 13,581 µm·s^−1^. Additionally, in the bubble formation region, the bubble reaches the velocity of the liquid almost instantaneously (i.e., homogeneous flow).

The tests were conducted with micropipettes of different internal diameters. Table 1 lists the micropipettes used, the manufacturer, the micropipette’s lengths (L) in millimeters and the micropipette’s internal diameter (*D*_p_) in micrometers. Some of the micropipettes used have Bezel shaped tips (e.g., MIC-SLM-30 and MIC-50-30). For these devices the internal diameter (e.g., 4.6 µm for MIC-SLM-30) and the largest ellipsoidal axis (e.g., 11 µm for MIC-SLM-30) are given.

The experiments were conducted using the presented emulsion with velocity in the range 11,035 µm·s^−1^ < U_l_ < 13,581 µm·s^−1^, considering a homogeneous flow (i.e., bubble quickly reaching the liquid velocity), hybrid device with several micropipettes with Reynolds number and capillary number in the ranges of 0.047 < Re < 0.058 and 0.0017 < Ca < 0.0021, respectively.

Additionally, we carried out experiments using the MIC-SLM-30-based device with higher air outlet pressure of 1.04 bar and higher liquid flow of Q_l_ = 1550 µL·min^−1^. In this case, Reynolds number, capillary number and liquid velocity were Re = 0.562, Ca = 0.0205 and U_l_ = 131,568 µm·s^−1^, respectively, using the same emulsion as in the previous experiments.

## 3. Results and Discussion

**Figure 3:**
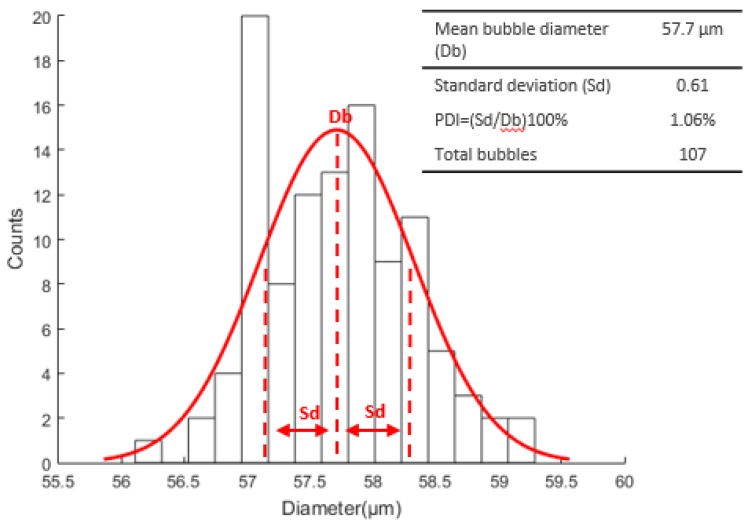
Microbubble diameter distribution curve using a MBB-BP-L-30 micropipette.

Tests were carried out and the micropipette models MIC-SLM-30, MSC-20-30, and MBB-BP-L-30 presented the best results. Using the MBB-BP-L-30 micropipette, we obtained an average bubble diameter of 57.7 µm in 8900 images captured at 150 fps and a total of 107 isolated microbubbles were detected in experiments, with the liquid phase flow rate at Q_l_ = 130 µL·min^−1^, gas phase flow rate at Q_g_ = 10.88 nL·min^−1^, Re = 0.047, Ca = 0.0017 and U_l_ = 11,035 µm·s^−1^. Figure 3 illustrates the diameter distribution curve for the analyzed bubbles considering the number of events occurred.

**Figure 4:**
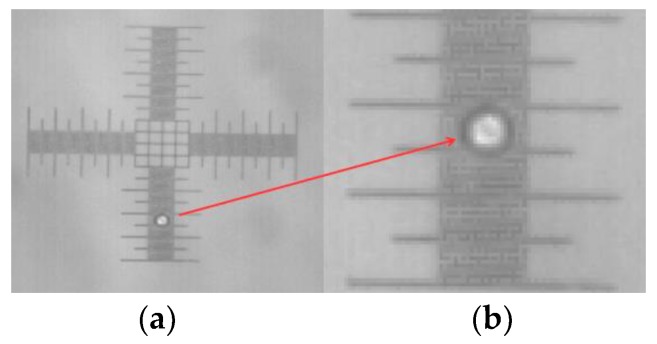
Microbubble generated with a MBB**-**BP-L-30 micropipette. (**a**) Microbubble over the 1DIV/10 μm micrometric ruler and (**b**) zoomed version of the microbubble region.

Figure 4 illustrates the microbubble generated by the MBB-BP-L-30 micropipette, using the digital camera coupled to a 4.3× magnification microscope and a 1 DIV/10 μm micrometer ruler scale. Figure 4a presents microbubbles over a 1 DIV/10 μm micrometer ruler and Figure 4b a zoomed version of the microbubble region.

**Figure 5:**
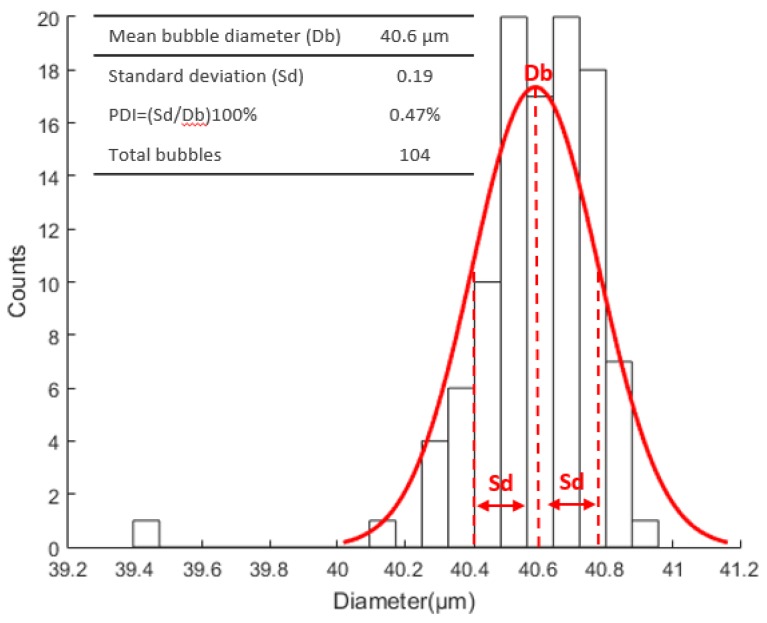
Microbubbles diameter distribution curve generated using the MSC-20-30 micropipette.

In the experiments using the MSC-20-30, we obtained an average bubble diameter of 40.6 µm in 6650 images captured at 100 fps and a total of 104 isolated microbubbles were detected in experiments, with the liquid phase flow rate being adjusted to Q_l_ = 160 µL·min^−1^, gaseous phase flow rate of Q_g_ = 3.28 nL·min^−1^, Re = 0.058, Ca = 0.0021, and U_l_ = 13,581 µm·s^−1^. Figure 5 presents the diameter distribution curve of microbubbles using MSC-20-30 micropipette.

**Figure 6:**
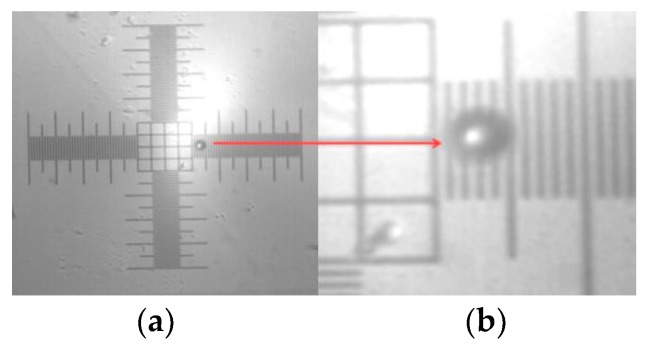
Microbubble image taken with a MSC-20-30 micropipette, microbubble over the 1 DIV/10 μm micrometric ruler and (**b**) zoomed version of the microbubble region.

Figure 6 is the image obtained using the MSC-20-30 micropipette using the same apparatus shown in Figure 4.

**Figure 7:**
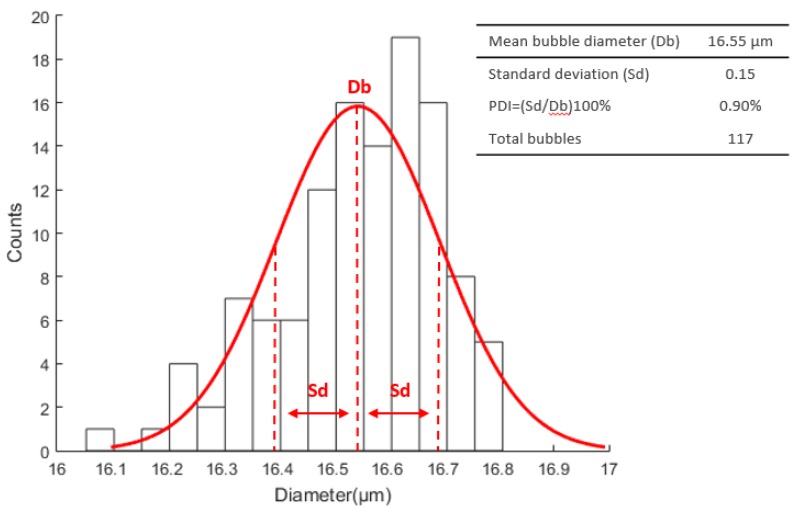
Microbubbles’ diameter distribution curve, using the MIC-SLM-30 micropipette.

Using the MIC-SLM-30 micropipette, we obtained an average bubble diameter of 16.6 µm in 15,120 images captured at 150 fps and a total of 117 isolated microbubbles were detected in experiments. The liquid phase flow rate was adjusted to Q_l_ = 130 µL·min^−1^, gas phase flow rate was adjusted to Q_g_ = 0.165 nL·min^−1^, Re = 0.047, Ca = 0.0017, and U_l_ = 11,035 µm·s^−1^. Figure 7 illustrates the microbubbles diameter distribution curve considering the number of events occurred.

**Figure 8:**
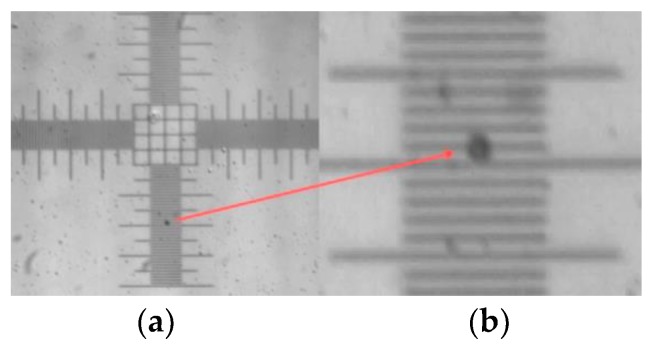
Microbubble image taken with a MIC-SLM-30 micropipette, (**a**) microbubble over the 1DIV/10 μm micrometric ruler and (**b**) enlarged microbubble image.

Using the same method illustrated in Figure 4, the microbubble image generated by MIC-SLM-30 micropipette was obtained, as can be seen in Figure 8; in which: (a) microbubbles over the 1 DIV/10 μm micrometer ruler and (b) enlarged microbubble image.

Table 2 lists the results obtained considering the mean diameter of the microbubble (*D*_b_), standard deviation (S_d_) and polydispersity index (PDI) using the MBB-BP-L-30, MSC-20-30, and MIC-SLM-30 micropipettes.

Different microbubble diameters, proportional to adjustments made in the gaseous and liquid phase, were obtained in experiments performed with the hybrid device and using the MIC-SLM-30 micropipette. Table 3 lists the resulting microbubble diameter mean, standard deviation, polydispersity index, number of microbubbles generated (NB), gaseous phase flow (Q_g_), and liquid phase flow (Q_l_) obtained in the experiments using the MIC-SLM-30 micropipette.

**Figure 9:**
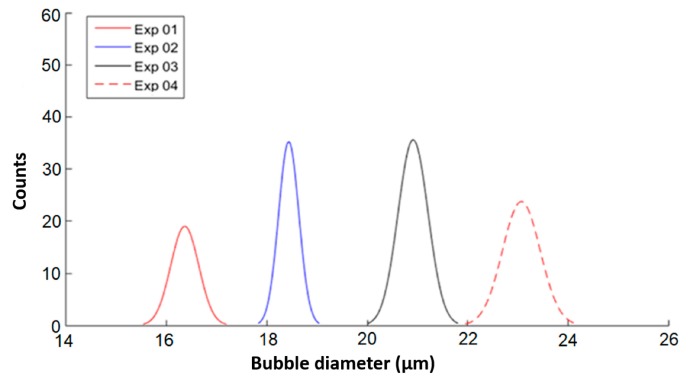
Gaussian distribution curve for microbubbles of different diameters due to liquid and gaseous phase flow variation.

Figure 9 illustrates the Gaussian distribution curves for the microbubbles diameter for the various experiments listed in Table 3.

**Figure 10:**
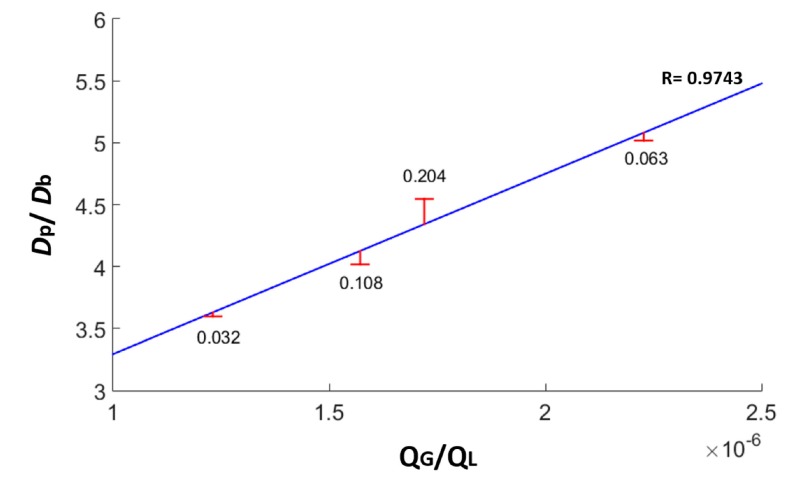
Microbubble production linearity in relation to gaseous and liquid phase flow. In which, *D*_b_ is the generated microbubbles’ mean diameter, *D*_p_ is the micropipette’s diameter (i.e., the diameter of the gas channel), Q_g_ is the gaseous phase flow rate, and Q_l_ is the liquid phase flow rate. The graph shows a linear trend with a Pearson correlation coefficient (R) of 0.9743.

The diameters of the generated microbubbles are linearly proportional to the liquid and gaseous flow rates variation. This shows that the proposed device is capable of generating different sized microbubbles with a high degree of uniformity. Figure 10 shows the microbubble production linearity in relation to the gaseous and liquid phase flow rate using a MIC-SLM-30 micropipette, with the absolute errors and the Pearson correlation coefficient (R) of 0.9743.

Table 4 presents the relation between the diameters of the microbubbles generated by the hybrid junction devices for MIC-SLM-30, MSC-20-30, and MBB-BP-L-30 micropipettes and the micropipette internal diameters.

A characteristic polynomial was defined (Equation (1)) using the Matlab computer simulation tool. This polynomial enables the estimation of the micropipette diameters capable of generating microbubbles of different diameters including those for clinical use (i.e., diameters smaller than 10 μm).

(1) $$D_{p}=0.0128\times {D_{b}}^{2}-0.0930\times D_{b}\;+\;2.6226$$

**Figure 11:**
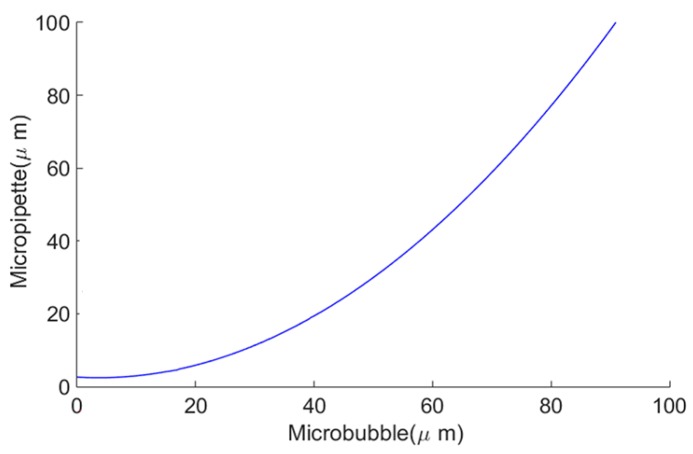
Estimation curve of the micropipette internal diameter as a function of the microbubble diameter.

Using this polynomial, a curve is shown in Figure 11 which estimates the micropipette’s internal diameter as a function of the desired microbubble diameter. According to the estimation curve presented in Figure 11, in order to obtain microbubbles smaller than 10 μm, the micropipette must have an internal diameter (*D*_p_) of less than 3.0 μm for the given gaseous and liquid phases parameters presented.

**Figure 12:**
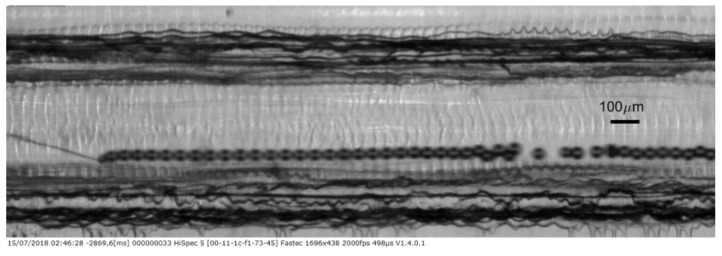
Process of bubble generation with the hybrid microfluidic device with hybrid microfluidic device, production rate of approximately 1800 s^−1^ bubbles. Gas pressure at 1.04 bar and liquid flow in 1.55 mL·min^−1^.

For the higher flow experiment using the device with the MIC-SLM-30 micropipette with air outlet pressure of 1.04 bar and higher emulsion flow of Q_l_ = 1550 µL·min^−1^, a video is presented with estimated bubble production of approximately 1800 bubbles per second (ESI†: microbubble_gen.avi). The 1696 × 438-pixel resolution video was acquired at 2000 fps but is shown at a slower frame rate to allow proper visualization. The Figure 12 presents one frame of this video.

**Figure 13:**
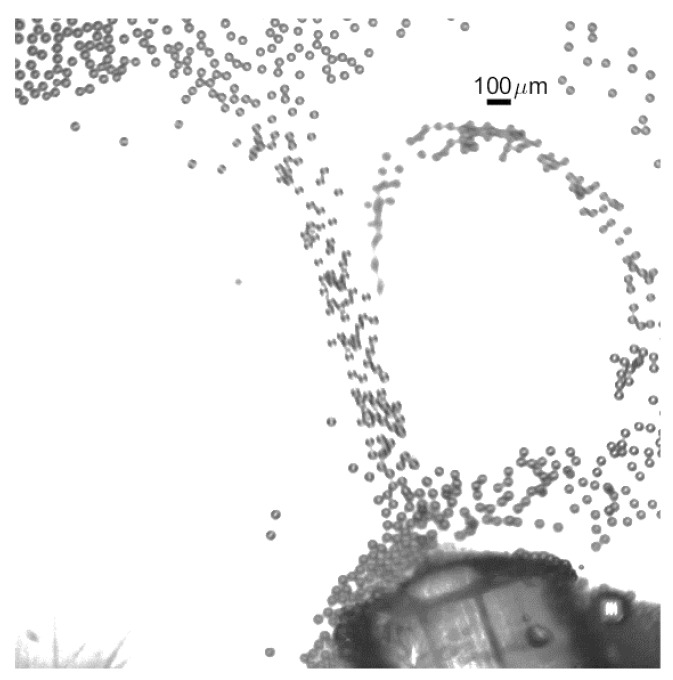
Bubbles acquired at the outlet of the hybrid device.

Bubbles were collected into a Petri dish as shown in the Figure 13. The images were recorded with the high frame rate camera attached to a microscope.

**Figure 14:**
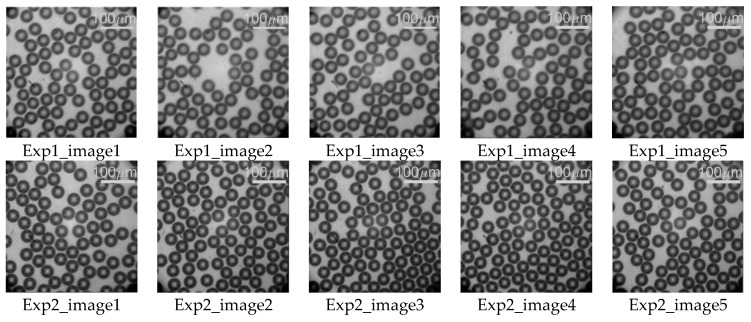
Images of microbubbles from each experiment performed.

Two independent experiments were conducted at the same gaseous and liquid parameters with images recorded along 4 s each with an approximate production of 7200 bubbles. The Figure 14 presents five images for each experiment. A Matlab-based script was developed such as the processed frames considered that no bubble in a given evaluated frame could be still visible in the following evaluated frame.

**Figure 15:**
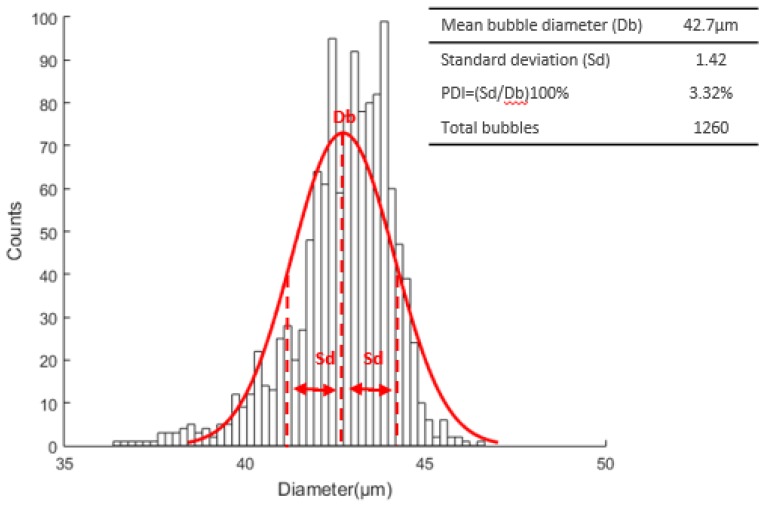
Microbubble diameter distribution curve with a mean diameter, standard deviation, and PDI of 42.7 μm, 1.42, and 3.32%, respectively.

For the first experiment, 1260 bubbles were measured resulting in a mean diameter, standard deviation and PDI of 42.7 μm, 1.42, and 3.32%, respectively. The Figure 15 presents the Gaussian distribution of the corresponding experiment.

**Figure 16:**
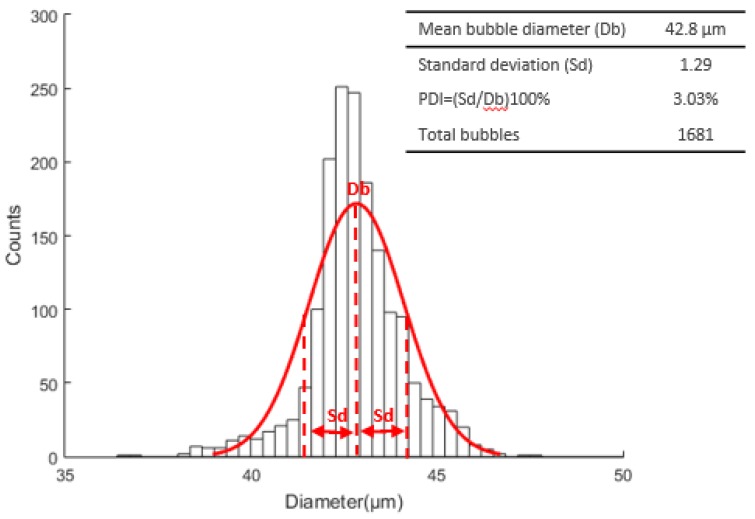
Microbubble diameter distribution curve with a mean diameter, standard deviation, and PDI of 42.8 μm, 1.29, and 3.03%, respectively.

For the second experiment, 1681 bubbles were measured resulting in a mean diameter, standard deviation and PDI of 42.8 μm, 1.29, and 3.03%, respectively. The Figure 16 presents the Gaussian distribution of the corresponding experiment.

**Figure 17:**
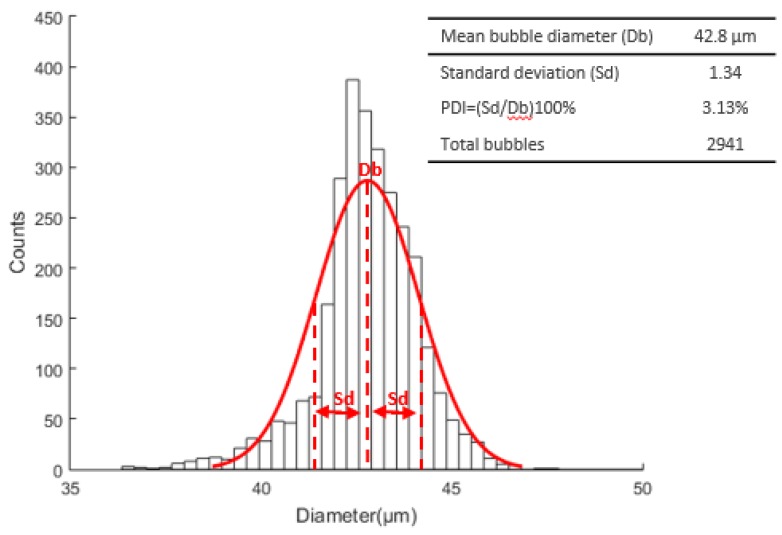
Microbubble diameter distribution curve with a mean diameter, standard deviation, and PDI of 42.8 μm, 1.34, and 3.13%, respectively.

The Figure 17 shows the overall 2941 bubbles measured in both experiments, resulting in a mean diameter, standard deviation, and PDI of 42.8 μm, 1.34, and 3.13%, respectively.

The use of 3D printing in the manufacturing process made it possible to develop a hybrid junction microfluidic device capable of generating monodisperse microbubbles coated with a lipid associated with surfactant in the order of 16.6 μm in diameter, which is close to the desired size for clinical applications. The hybrid device was able to produce microbubbles with a high degree of uniformity with a 0.91% polydispersity index. In the experiments using different micropipette models microbubbles with polydispersity indexes between 0.47% and 1.06% were generated. These values are close to the 1% index obtained by Stride and Edirisinghe [10]. The relation of the generated microbubble diameter as a function of the internal diameter of the micropipettes was experimentally defined and a characteristic curve relating these parameters through the use of a 2nd order polynomial was elaborated and shown in Equation (1).

One of the formats of the needles used in this work were Bezel shape tip model MIC-SLM-30. In our understanding the shape of the needle has an important connection with the formation of the microbubble, because it has direct implication in the cross section, the shear stress of the fluid, as well as in the stability of microbubble production.

In T-junction systems according to Garstecki and Fuerstman (2006), the equation in the convergence region of the flows can be described according to Equation (2) [17].

(2) $$\frac{L}{d}=1+\alpha \frac{Q_{g}}{Q_{l}}$$

where L is the diameter of the microbubble, d represents the width of the channel in the gas phase, $Q_{g}$ and $Q_{l}$ are respectively the flow rates of the gas and liquid phases of the fluids and α is a constant. Note that by changing the shape of the needle, we change $D_{m}$ and consequently the conditions for the formation of the microbubble. The proportionality constant for the above equation depends on the geometric characteristics of the device (e.g., channel profile), but is almost independent of the fluid properties for Re << 1 [17]. All the needles used in this work have a slope of 30° with various diameters.

The obtained equation was evaluated in two other studies using the lipid matrix as the microbubble coating and air for the gaseous phase. Araujo Filho et al. (2012), conducted experiments using air encapsulated by a lipid matrix and Tween 80 surfactant for generating microbubbles. The mean microbubble diameter obtained was 73.74 μm using a 70 μm internal diameter micropipette. Using Equation (1), the micropipette diameter would be 65.36 μm, with an absolute error of 4.64 μm and a 6.62% percentage error [25]. For the second experiment, conducted by Duncan and Needham (2004), the microbubble with an initial diameter of 15 μm was produced and maintained at the tip of a 4 μm micropipette. Using Equation (1) for this case, the micropipette diameter would be 4.11 μm, with an absolute error of 0.11 μm and a 2.75% percentage error [27]. Therefore, the polynomial obtained in this work seems to be applicable to other studies resulting in small percentage error for the estimations.

The estimations obtained from the application of Equation (1) to the referred studies allows to determine the approximated micropipette internal diameter necessary to generate a desired microbubble size, and vice versa. The mathematically allowed point (0; 2.6226), clearly has no real significance, since it is not possible to produce 0-μm microbubbles. Therefore, it is a forbidden point, as are the negative values for *D*_b_. Since Equation (1) is a continuous function for any value of *D*_b_ and we are interested only in values that are physically possible, the characteristic polynomial’s general solution is valid for *D*_b_ > 0 being structured as following: $S=\{\mathrm{for}\;\mathrm{every}\;D_{b}\in ℜ/D_{b}>0$}. The 3D printing technique enabled the development of the hybrid device (microfluidic device with inserted micropipette) costing approximately US$40. The microfluidic device with the MIC-SLM-30 micropipette allowed the generation of microbubbles with an average diameter of 16.6 μm. According to Equation (1), micropipettes with inner diameters below 3 μm would allow the production of microbubbles of less than 10 μm of diameter. Recently, Word Precision Instruments started selling micropipettes of inner tip diameters down to 0.1 μm. The use of these micropipettes in the hybrid devices will certainly allow the production of microbubbles smaller than 10 μm. In addition, according to Jiang et al. (2016), the diameter of the microbubbles can be further reduced with the use of multiple T-junctions, over there from also increasing the production of microbubbles [3]. According to the Eptein–Plesset equation, the stability of the microbubble can be improved by using gases with high molecular weight and low water solubility. An example of such gas is the perfluorobutane whose molecular weight is 238.03 g·mol^−1^. As an example, this gas could be used for clinical applications to increase the survival of the micro bubbles due to their resistance to penetration in the water, combined with the difficulty of gas exchange between the gas core and the environment external to the microbubble.

Additionally, the rate of bubble production per second needed will be application dependent. At the current state of this work we are capable, for example, of producing around 1800 microbubbles per second, with mean diameter of 42.8 μm. This would allow us having a surface of 5754.9 μm^2^ for each bubble resulting in a 10.36 mm^2^ of available bubble surface per second. If clinical application is considered, the bubble diameter need to be reduced, therefore reducing the available bubble surface. For the new bubble surface obtained one needs to consider the amount of drug could be carried in such production and latter correlate the amount of drug carried with the amount of drug locally required to be delivered.

Besides understanding the dimension and flow relations for microbubble production, another aim of this study was to show the manufacturing process of microfluidic devices using the 3D printing technique, wherein the device is manufactured in a single step, eliminating additional procedures such as adapting connections and assemblies and adding the potential to produce circular sections. In these microfluidic devices, commercial micropipettes were inserted for the generation of monodisperse microbubbles. This technique is a simple manufacturing model compared to the technique of manufacturing microfluidic devices by soft lithography. Soft lithography requires several steps in the production of the devices, needing expensive and dedicated equipment in each stage of production, in addition to the demand for very clean environments in the manufacturing of the devices [28]. A potential disadvantage is that micro channel obstruction might occur in microfluidic devices due to several factors (e.g., emulsion impurities and emulsion drying in the channels). On the other hand, in these cases, new devices can be manufactured simply and economically.

## 4. Conclusions

This paper presented the lipid coated microbubble generation process using a low-cost and simple 3D deposition manufacturing processes associated with commercial micropipettes inserted in microfluidic devices at an approximate cost of USD40. Microbubbles with an average size of 16.6 μm to 57.7 μm and a PDI between 0.47% and 1.06% were generated. For the experiment at higher bubble rate, the average diameter was 42.8 μm, with increased PDI of 3.13%, leading to a conclusion that with this hybrid device, the PDI increases in a higher bubble production rate due to the proximity of the tip with the outlet channel wall, where the liquid flow is more unstable. In addition, a linear relationship between Q_g_/Q_l_ and *D*_b_/*D*_p_ was found, as well as a polynomial equation was found that seems useful to estimate micropipette internal diameter necessary to generate a desired microbubble size, and vice versa. For applications such as drug carriers, smaller microbubbles are necessary. The results show the potential of such microbubble production in case micropipettes with internal diameter of less than 3 μm are used.

## Figures and Tables

**Table 1:** Micropipettes used in the generation of microbubbles.

Model	L (mm)	*D*_p_ (µm)	Manufacturer
MIC-SLM-30 *	55	11 and 4.6	Origio
MIC-50-30 *	55	9 and 5.3	Origio
MSC-20-30	57	20	Origio
MAH-SM-30	57	9	Origio
MBB-BP-L-30	57	40	Origio

* Bezel shaped tip.

**Table 2:** Results obtained using the various micropipettes.

Micropipette Model	MBB-BP-L-30	MSC-20-30	MIC-SLM-30
Diameter microbubble (*D*_b_)	57.7 µm	40.6 µm	16.6 µm
Standard deviation (S_d_)	0.61	0.19	0.15
Polydispersity index (PDI)	1.06%	0.47%	0.90%

**Table 3:** Microbubble diameter variation according to gaseous and liquid phase flow rates using the MIC-SLM-30 micropipette.

Test n°	*D*_b_ (µm)	S_d_	PDI (%)	NB	Q_l_ (µL·min^−1^)	Q_g_ (nL·min^−1^)
Exp 01	16.6	0.15	0.91	117	130	0.16
Exp 02	18.5	0.23	1.24	169	70	0.11
Exp 03	21.0	0.30	1.43	208	250	0.43
Exp 04	23.0	0.37	1.60	163	220	0.49

**Table 4:** Diameter of the microbubbles (*D*_b_) generated in relation to the internal diameter of the micropipettes (*D*_p_).

Modelo	*D*_p_ (µm)	*D*_b_ (µm)
MIC-SLM-30	11 and 4.6	16.6
MSC-20-30	20	40.6
MBB-BP-L-30	40	57.7

## Supplementary Materials

The following are available online at http://www.mdpi.com/2072-666X/9/8/387/s1, Video: S1_Video Experimental apparatus.mpg and microbubble_gen.avi, File: microbubble_gen.video_information.txt.
